# Autoantibodies against Phospholipase A_2_ Receptor in Korean Patients with Membranous Nephropathy

**DOI:** 10.1371/journal.pone.0062151

**Published:** 2013-04-26

**Authors:** Yun Jung Oh, Seung Hee Yang, Dong Ki Kim, Shin-Wook Kang, Yon Su Kim

**Affiliations:** 1 Department of Internal Medicine, Seoul National University Hospital, Seoul, Korea; 2 Department of Internal Medicine, Yonsei University College of Medicine, Seoul, Korea; 3 Seoul National University Kidney Research Institute, Seoul, Korea; 4 Severance Biomedical Science Institute, Brain Korea 21, Yonsei University, Seoul, Korea; Centro di Riferimento Oncologico, IRCCS National Cancer Institute, Italy

## Abstract

The data were presented in abstract form at the 45^th^ meeting of the American Society of Nephrology, October 30-November 04 2012, San Diego, CA, USA. Circulating autoantibodies against M-type phospholipase A_2_ receptor (PLA_2_R) are important pathogenic antibodies of idiopathic membranous nephropathy (MN) in adults. However, previous studies on the clinical impact of anti-PLA_2_R antibodies demonstrated several limitations, including insufficient numbers of study subjects and different time points and methods for anti-PLA_2_R antibody measurement. To verify the clinical significance of anti-PLA_2_R antibodies in Korean patients with MN, we measured autoantibodies in serum samples obtained at the time of biopsy from a total of 100 patients with idiopathic MN who had not yet received immunosuppressive treatment. We detected anti-PLA_2_R antibody in 69 patients, and we observed that autoantibody reactivity reflected the severity of disease activity. Proteinuria and hypoalbuminemia were more severe in patients with anti-PLA_2_R than in those without the autoantibodies (2.95 g/g vs. 6.85 g/g, *P* = 0.003; 3.1 g/dL vs. 2.5 g/dL, *P* = 0.004, respectively). Additionally, the clinical severities worsened proportionally as the levels of anti-PLA_2_R antibodies increased (*P* = 0.015 and *P for trend* <0.001 for proteinuria and hypoalbuminemia, respectively). However, neither the levels nor the presence or absence of anti-PLA_2_R antibody showed a significant correlation with clinical outcomes, such as remission rate and time to remission. In conclusion, we observed that anti-PLA_2_R antibodies are highly prevalent in Korean patients with idiopathic MN and that they reflect the clinical disease activity before the administration of immunosuppressive treatment. However, the levels of anti-PLA_2_R antibody at the time of kidney biopsy may not predict the clinical outcomes in current clinical practice.

## Introduction

Membranous nephropathy (MN) constitutes a major glomerulonephritis that causes nephrotic syndrome in adults [1]. The in situ formation of immune complexes in the subepithelial space of the glomerular basement membrane is the distinct pathologic feature of MN [2], [3], [4]. Recently, M-type phospholipase A_2_ receptor (PLA_2_R) was identified as the target antigen of autoantibodies in adults with idiopathic MN [5]. Several studies have reported that autoantibodies against PLA_2_R not only have a direct pathogenic role but also serve as sensitive and specific markers for idiopathic MN [5], [6], [7], [8], [9]. However, there have been discrepancies in results regarding the relationship between anti-PLA_2_R levels and the clinical presentation [6], [7], [8], [10], [11]. There may be several reasons for these discrepancies. First, previous studies have reported that the time point of anti-PLA_2_R measurement was different [5], [7], [11]. Comparing data measured at different stages of MN could lead to an inappropriate interpretation. Second, the methods for detecting and titrating anti-PLA_2_R were different among the studies [6], [8], [11]. Third, most of the previous studies did not include a sufficient number of patients [5], [6], [8]. Additionally, the correlation between autoantibodies and the clinical status had not been thoroughly investigated in Asian patients with MN, despite the hypothesis that race or ethnic background might be associated with the incidence or prognosis of the disease [12], [13], [14], [15], [16].

In our study, we measured anti-PLA_2_R levels at the time of kidney biopsy in a large number of patients with idiopathic MN who had not received immunosuppressive treatment before kidney biopsy, and we explored the relationship between anti-PLA_2_R and disease activity and clinical outcomes.

## Materials and Methods

### Study subjects and serum samples

We conducted our study with the approval of the institutional review boards of Seoul National University Hospital and Yonsei University Severance Hospital in Seoul, Korea. All of the participants provided their written informed consent according to the Declaration of Helsinki. The ethics committees of Seoul National University Hospital and Yonsei University Severance Hospital approved this study. We evaluated MN patients with biopsy-proven MN who were diagnosed between 2002 and 2011. In this study, we included 100 idiopathic and 9 secondary MN patients whose serum samples were collected at the time of kidney biopsy. To compare the prevalence of anti-PLA_2_R between patients with active-phase MN and patients in remission, we included 19 additional patients with idiopathic MN who had entered remission. Additionally, we used serum specimens from 14 healthy volunteers as normal controls.

We confirmed the diagnosis of idiopathic MN by histologic findings, including subepithelial electron-dense deposits on electron microscopy and a diffuse granular pattern of IgG and C3 staining on immunofluorescence microscopy. We diagnosed secondary MN in patients who had a suggestive cause of MN, including hepatitis B virus (HBV), hepatitis C virus (HCV), lupus, and malignancy, with comparable pathologic features of MN.

### Clinical data

We collected the clinical information on disease severity, treatment, remission, and relapse by reviewing the subjects’ medical records. We categorized our subjects into three groups according to their risk for disease progression based on clinical characteristics [17], [18], [19]. Low risk was defined as urine protein-creatinine ratio (uPCR) less than 4.0 g/g and estimated glomerular filtration rate (eGFR) ≥60 mL/min/1.73 m^2^ (calculated using the original Modification of Diet in Renal Disease equation [20], which is most commonly used and validated in Koreans [21]). Moderate and high-risk patients had uPCR between 4.0–8.0 g/g and eGFR ≥60 mL/min/1.73 m^2^ and uPCR exceeding 8 g/g and impaired renal function (eGFR<60 mL/min/1.73 m^2^), respectively. Remission was defined as proteinuria reduction of 50% or greater from baseline and uPCR less than 3.5 g/g. Relapse was defined as uPCR >3.5 g/g after some period of remission.

### Human glomerular extracts

Normal renal cortexes, which were part of the radical nephrectomy specimens from patients with renal cell carcinoma, were obtained with institutional review board approval. We extracted human glomerular protein as previously described [5]. We isolated the glomeruli from the kidney with graded sieving, using 100-, 125-, and 150-µm-sized meshes. The glomerular pellet was homogenized in an equal volume of RIPA buffer (150 mM NaCl, 1% Triton- X-100, 1% deoxicholic acid sodium salt, 0.1% SDS, 50 mM Tris-HCl, pH 7.5, 2 mM EDTA; GenDEPOT, Barker, TX, USA) with complete protease inhibitor cocktail (Roche Applied Science, Indianapolis, IN, USA) on ice. The glomerular homogenate was centrifuged twice at 12,000 rpm for 20 minutes at 4°C, and the supernatant was isolated. To remove the contaminating human IgG, we incubated the human glomerular extracts (HGEs) with Pure down Protein G-Agarose (GenDEPOT, Barker, TX, USA). We proved that we had removed contaminating human IgG by confirming that there was no detectable band in immunoblotting with anti-human IgG.

### Western blotting

Equal amounts of HGEs were loaded onto 6% SDS-polyacrylamide gels (PAGE) under nonreducing conditions and transferred to Immobilon-FL 0.4 µM polyvinylidene difluoride membranes (Millipore, Bedford, MA, USA). Membranes were incubated in 5% blocking buffer containing 2% bovine serum albumin, and human serum was initially used at a dilution of 1∶100 as the primary Ab. For samples with negative results at a dilution of 1∶100, we performed western blotting at a dilution of 1∶25 and confirmed non-reactivity based on the negative results with the higher concentration of Ab. The serum samples with positive results were retested at gradually increased dilutions up to 1∶8000. Sheep anti-human IgG4 (The Binding Site, Birmingham, UK) antibodies were used as a secondary Ab at a dilution of 1∶3000, followed by peroxidase-conjugated donkey anti-sheep IgG (The Binding Site, Birmingham, UK) antibodies as the detecting Ab at a dilution of 1∶10000. A goat polyclonal Ab against PLA_2_R (Sigma-Aldrich, St. Louis, MO, USA) was used at a dilution of 1∶400 to confirm the location of the PLA_2_R band. The membranes were incubated with chemiluminescent substrate (Pierce ECL Plus Western Blotting Substrate, Thermo, Rockford, IL, USA) for 1 minute and exposed to HyBlot CL autoradiography film (Denville Scientific, Inc. Metuchen, NJ, USA). The exposure times were typically 30 to 60 seconds for positive bands, and extended up to 10 minutes for weak or negative bands. The labeled proteins were detected by the enhanced chemiluminescence system (ECLTM PRN 2106; Amersham Pharmacia Biotech, Buckinghamshire, UK). To explore the correlation between clinical status severity and anti-PLA_2_R levels, we categorized the patients into 4 groups according to anti-PLA_2_R levels. Group 1 (Negative) included patients without anti-PLA_2_R, and group 2 (1∶100 (+)) included patients whose serum samples reacted with PLA_2_R at a dilution of 1∶100. Patients whose serum reacted with PLA_2_R at increased dilutions up to 1∶2000 were categorized in group 3 (1∶2000 (++)), and the other patients with positive results between 1∶2000 and 1∶8000 dilution were categorized as group 4 (1∶8000 (+++)).

### Statistical analysis

For data description, continuous variables with symmetric distribution were presented as the mean±SD, and non-normally distributed variables were expressed as medians (25–75% interquartile range). Student’s *t*- test and analysis of variance (ANOVA) were used for parametric analysis, and ANOVA with polynomial contrast was performed for a trend analysis. The Mann-Whitney *U* test and Kruskall-Wallis test were used for nonparametric analysis. The Dunn method was performed for multiple comparisons in nonparametric analysis. Categorical variables were described as frequencies or percentages, and the data were analyzed with χ^2^ tests. The four groups were compared using the Mantel-Haenszel *χ^2^* tests for dichotomized variables. All of the statistical analyses were conducted using SPSS, version 19.0 (Chicago, IL, USA).

## Results

### Clinical characteristics of idiopathic MN

Table 1 shows the clinical characteristics of idiopathic MN patients at the time of kidney biopsy. The average age was 54.7±13.9 years. The mean serum creatinine and eGFR were 0.91±0.35 mg/dL and 90±28 mL/min/1.73 m^2^, respectively. The mean serum albumin level was 2.7±0.7 g/dL, and the median uPCR was 6.07 g/g. None of the patients received immunosuppressive treatment before kidney biopsy.

**Table 1:** Clinical characteristics of patients with idiopathic MN at the time of kidney biopsy according to anti-PLA_2_R reactivity.

	Total	Anti-PLA_2_R(+)	Anti-PLA_2_R(-)	*P* value
Number of patients	100	69	31	
Age (years)	54.7±13.9	55.1±12.7	53.8±16.5	0.697
Male	51 (53.0%)	40 (58.0%)	13 (41.9%)	0.137
Diabetes mellitus	12 (12.0%)	6 (8.7%)	6 (19.4%)	0.182
Hypertension	42 (42.0%)	26 (37.7%)	16 (51.6%)	0.192
Total cholesterol (mg/dL)	271±99	278±101	255±94	0.288
Serum creatinine (mg/dL)	0.91±0.35	0.91±0.35	0.90±0.35	0.979
eGFR (mL/min/1.73 m^2^)	90±28	91±25	91±35	0.921
Serum albumin (g/dL)	2.7±0.7	2.5±0.6	3.1±0.9	0.004
uPCR (g/g)	6.07 (3.17–9.86)	6.85 (4.87–9.98)	2.95 (1.14–9.09)	0.003
uPCR >3.5 g/g	75 (75.0%)	60 (87.0%)	15 (48.4%)	<0.001
RAS blocker	81 (81.0%)	58 (84.1%)	23 (74.2%)	0.245
Statin	85 (85.0%)	61 (88.4%)	24 (77.4%)	0.224
Anti-coagulation	5 (5.0%)	5 (7.2%)	0 (0%)	0.320

The data are expressed as the mean±SD or median (25–75% interquartile range). MN, membranous nephropathy; Anti-PLA_2_R, anti-phospholipase A2 receptor antibody; eGFR, estimated glomerular filtration rate calculated using the Modification of Diet in Renal Disease formula; uPCR, urine protein-creatinine ratio; RAS blocker, renin angiotensin systemic blocker.

### Prevalence of anti-PLA_2_R

**Figure 1:**
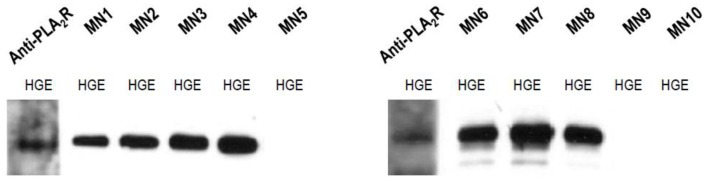
Representative western blot of anti-PLA_2_R in the serum of patients with idiopathic MN. Human glomerular extracts were electrophoresed, and serum samples of idiopathic membranous nephropathy patients were used as the primary antibody at a dilution of 1∶100. The band detected by immunoblotting with commercial anti-PLA_2_R antibody was used to indicate the position of PLA_2_R. Serum samples from a number of patients with idiopathic MN (except MN5, MN9, and MN10) and the commercial anti-PLA_2_R antibody demonstrated positive bands at approximately 185kD.

We examined anti-PLA_2_R reactivity with western blotting and confirmed the presence of a 185 kD protein band using native antibody (Ab) against human PLA_2_R Ab and serum samples from patients with MN (Figure 1). A total of 69 of 100 patients with idiopathic MN had autoantibodies against PLA_2_R at the time of kidney biopsy. The prevalence of anti-PLA_2_R was 80.0% (60 of 75) in patients with idiopathic MN and nephrotic range proteinuria at the time of biopsy. The autoantibody was not detected in any of the healthy control subjects (n = 14) (data not shown).

We compared anti-PLA_2_R reactivity between patients at the time of kidney biopsy (n = 100) and another subset of patients in remission after treatment (n = 19). Whereas a large number of patients with idiopathic MN had autoantibodies against PLA_2_R at the time of diagnosis, the prevalence of anti-PLA_2_R reactivity was lower (15.8% vs. 69.0%) in patients who went into remission after treatment.

The causes of secondary MN were HBV (n = 6), HCV (n = 1), malignancy (n = 2), and systemic lupus erythematosus (n = 1). Anti-PLA_2_R reactivity was considerably lower in patients with secondary MN compared with patients with idiopathic MN. Two patients (1 with HBV and 1 with malignancy) showed reactivity against PLA_2_R. Immunostaining for IgG4 was observed in both patients with anti-PLA_2_R reactivity but it was not present in patients with secondary MN without anti-PLA_2_R, except in one patient who showed equivocal IgG4 staining (Table 2).

**Table 2:** Histologic findings of patients with secondary MN.

Patient	Cause of secondary MN	Anti-PLA_2_R	Subepithelial deposits	Subendothelial deposits	IgG4 deposits	Mesangial deposits	C1q deposits
S1	Malignancy	+	+	–	+	+	–
S2	HBV	+	+	–	+	+	–
S3	HBV	–	+	–	–	+	+
S4	HBV	–	+	–	–	–	+
S5	HBV	–	+	+	±	–	+
S6	HBV	–	+	–	–	–	+
S7	HCV	–	+	+	–	–	+
S8	Lupus	–	+	+	–	+	+
S9	Malignancy	–	+	–	–	–	–

MN, membranous nephropathy; Anti-PLA_2_R, anti-phospholipase A2 receptor antibody; HBV, hepatitis B virus; HCV, hepatitis C virus.

### Relationship between clinical status and anti-PLA_2_R reactivity/levels

In patients with idiopathic MN, we found significant correlations between anti-PLA_2_R reactivity and clinical parameters, including serum albumin and proteinuria (Table 1). Proteinuria was more severe in patients with anti-PLA_2_R compared with those patients without anti-PLA_2_R (uPCR, 6.85 g/g vs. 2.95 g/g, *P* = 0.003). The initial serum albumin levels were significantly lower in patients with anti-PLA_2_R than in patients without anti-PLA_2_R (2.5 g/dL vs. 3.1 g/dL, *P* = 0.004). The proportion of patients with nephrotic range proteinuria was significantly higher in the anti-PLA_2_R-positive group than in the negative group (87.0% vs. 48.4%, *P*<0.001). However, there was no significant difference in renal function between the groups.

**Figure 2:**
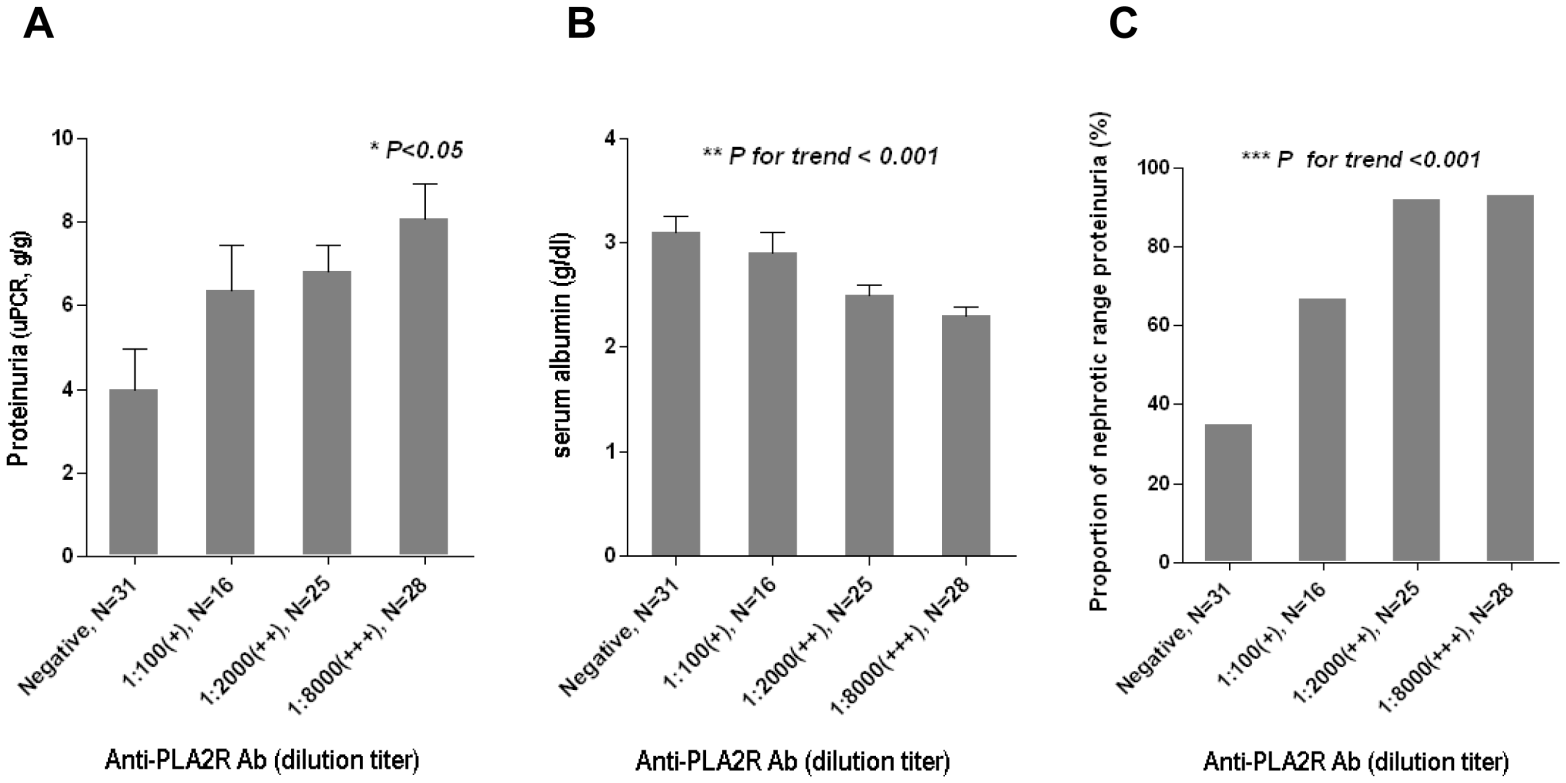
Relationship between anti-PLA_2_R levels and clinical parameters in patients with idiopathic MN. A: Proteinuria was more severe in patients with higher anti-PLA_2_R level. *Dunn’s multiple pairwise comparison test after Kruskal-Wallis test. B Severity of hypoalbuminemia was increased depending on the anti-PLA_2_R levels. **ANOVA test with polynomial contrast. C: The proportion of patients with nephrotic range proteinuria was increased according to the anti-PLA_2_R levels. *** Mantel-Haenszel χ^2^ test.

**Figure 3:**
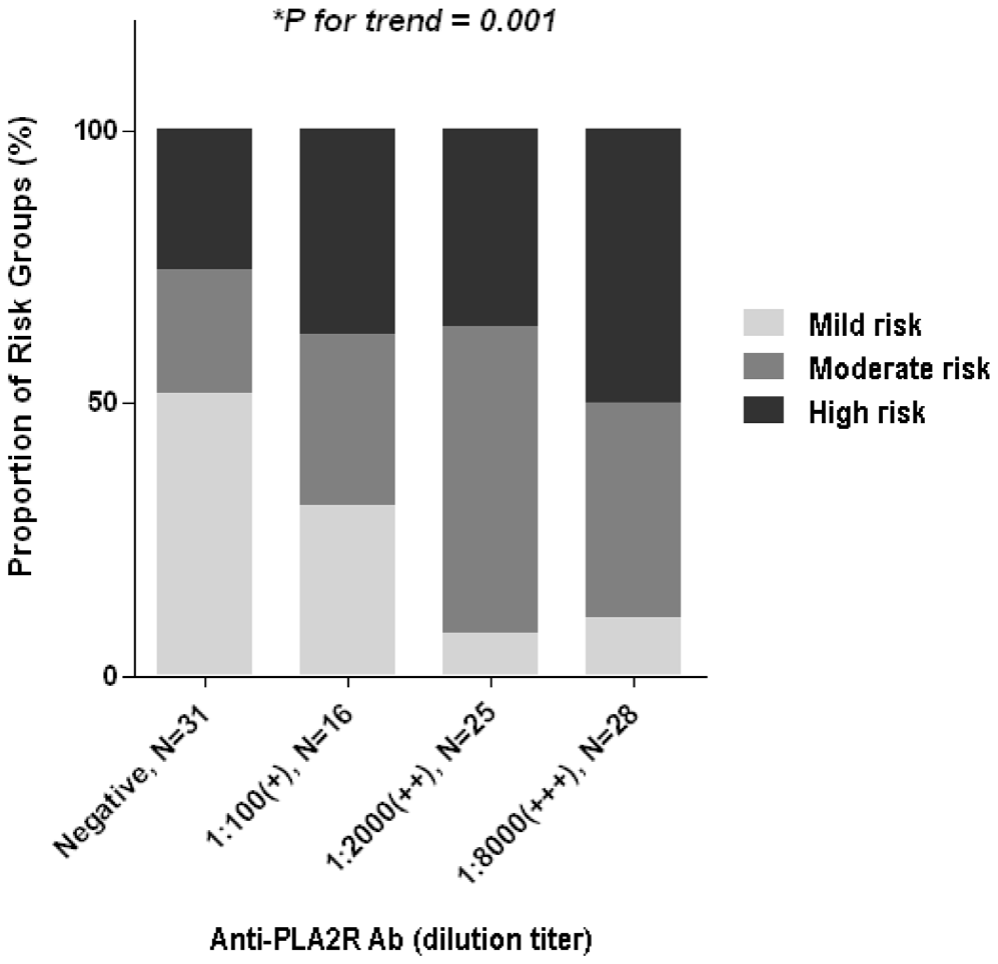
Proportion of patients categorized by risk for progression according to anti-PLA_2_R levels. The proportion of the high-risk group was gradually increased as anti-PLA_2_R levels increased. * Mantel-Haenszel χ^2^ test.

To investigate whether clinical disease activities were correlated with quantitative levels of anti-PLA_2_R, we compared clinical parameters among groups categorized by anti-PLA_2_R levels. As indicated in Figure 2, proteinuria and hypoalbuminemia became more severe as the anti-PLA_2_R levels increased. The proportion of patients with nephrotic range proteinuria increased gradually as the anti-PLA_2_R levels increased. Moreover, there was a significant difference in the proportion of groups stratified by risk for disease progression (Figure 3). The proportion of the high-risk group was the most dominant in the group with the highest anti-PLA_2_R levels, and the reverse was observed in the lowest group.

### Relationship between clinical outcomes and anti-PLA_2_R reactivity/levels

To address whether anti-PLA_2_R could predict the clinical outcomes of patients with idiopathic MN, we analyzed the clinical data of 77 of the 100 patients with idiopathic MN who had been followed up after kidney biopsy and had available clinical information, including details on treatment response and relapse. There has been no patient, in whom an initial diagnosis of idiopathic MN was reclassified as secondary MN as a result of the belated development of a secondary cause, such as malignancy during the follow-up period. The median follow-up time was 30 months, and 67.5% of the patients received immunosuppressive treatment (Table 3). There was no significant difference in the rate of receiving immunosuppressive treatment between the anti-PLA_2_R positive and negative groups. The second-line therapy was used in 4 patients, 2 of them went into remission after administration of second-line therapy. During the treatment, 6 patients developed steroid-induced. Additionally, in the majority of cases, infections comprised the clinically significant adverse reactions associated with immunosuppressive drugs; 5 patients received hospital treatment for severe infection, and one patient stopped immunosuppressive therapy because of a severe recurring infection. A total of 81.8% patients entered remission, and the median time to remission was 2.0 months. However, neither the remission rate nor the time to remission was significantly different between patients with and without anti-PLA_2_R. Furthermore, these clinical outcomes were not different among groups with different levels of anti-PLA_2_R (Table 4). Additionally, during the follow-up period, two patients reached doubling of serum creatinine levels compared to baseline, and one patient had a high titer of anti-PLA_2_R and the other exhibited a negative anti-PLA_2_R reactivity. Only one patient without anti-PLA_2_R progressed to end-stage renal disease.

**Table 3:** Clinical outcomes and general characteristics of patients with idiopathic MN according to anti-PLA_2_R reactivity.

		Total	Anti-PLA_2_R(+)	Anti-PLA_2_R(-)	*P* value
Number of patients		77	56	21	
Age		55±13.9	55±13.1	58±15.9	0.438
Male		40 (51.9%)	31 (55.4%)	9 (42.9%)	0.328
Total cholesterol (mg/dL)		281±99	286±105.1	265±83.4	0.397
Serum creatinine (mg/dL)		0.92±0.35	0.90±0.33	0.97±0.40	0.447
eGFR (mL/min/1.73 m^2^)		89±29	90±24.3	85±39.4	0.527
Serum albumin (g/dL)		2.5±0.6	2.5±0.5	2.8±0.7	0.036
uPCR (g/g)		6.80 (4.80–9.98)	6.87 (4.97–9.99)	5.35 (2.29–9.90)	0.106
uPCR >3.5 g/g		66 (85.7%)	52 (92.9%)	14 (66.7%)	0.007
RAS blocker		64 (83.1%)	48 (85.7%)	16 (76.2%)	0.325
Statin		69 (89.6%)	51 (91.1%)	18 (85.7%)	0.676
Anti-coagulation		4 (5.2%)	4 (7.1%)	0 (0%)	0.570
Immunosuppressive treatment		52 (67.5%)	38 (67.9%)	14 (66.7%)	0.921
Steroid		18 (34.6%)	14 (36.8%)	4 (28.6%)	
Steroid + Cyclosporine		14 (26.9%)	8 (21.1%)	6 (42.8%)	
Steroid + Tacrolimus		2 (3.8%)	2 (5.3%)	0 (0%)	
Steroid + Cyclophosphamide		16 (30.8%)	12 (31.6%)	4 (28.6%)	
Steroid + Mycophenolate mofetile		2 (3.8%)	2 (5.3%)	0 (0%)	
Remission rate		63 (81.8%)	45 (80.4%)	18 (85.7%)	0.746
Treatment-induced		46 (73.0%)	33 (73.3%)	13 (72.2%)	
Spontaneous		17 (27.0%)	12 (26.7%)	5 (27.8%)	
Time to remission (months)		2.0 (1.0–4.0)	2.0 (1.0–4.0)	2.0 (1.0–5.5)	0.580
Relapse		7 (9.1%)	5 (8.9%)	2 (9.5%)	1.000

The data are expressed as the number (%) or median (25–75% interquartile range) or the mean±SD. MN, membranous nephropathy; Anti-PLA_2_R, anti-phospholipase A_2_ receptor antibody. eGFR, estimated glomerular filtration rate calculated using the Modification of Diet in Renal Disease formula; uPCR, urine protein-creatinine ratio; RAS blocker, renin angiotensin systemic blocker.

**Table 4:** Clinical outcomes of patients with idiopathic MN according to anti-PLA_2_R levels.

Clinical outcomes		Anti-PLA_2_R levels				
		0 (Negative), N = 21	1∶100 (+)^a^, N = 11	1∶2000 (++)^b^, N = 22	1∶8000 (+++)^c^, N = 23	P value
Remission		18 (85.7%)	10 (90.9%)	16 (72.7%)	19 (82.6%)	0.540
Treatment-induced		13 (61.9%)	5 (45.5%)	13 (59.1%)	15 (65.2%)	
Spontaneous		5 (23.8%)	5 (45.5%)	3 (13.6%)	4 (17.4%)	0.380
Time to remission (months)		2.0 (1.0–5.5)	3.0 (1.3–4.0)	1.5 (1.0–4.8)	2.0 (1.0–5.3)	0.895

MN, membranous nephropathy; Anti-PLA_2_R, anti-phospholipase A_2_ receptor antibody; N, number of patients.

a Group of patients whose serum samples showed anti-PLA_2_R reactivity at dilutions of 1∶25 or 1∶100 with negative results at dilutions over 1∶100; ^b^Group of patients whose serum samples showed anti-PLA_2_R reactivity at dilutions of 1∶500 or 1∶2000 with negative results at dilutions over 1∶2000; ^c^Group of patients whose serum samples showed anti-PLA_2_R reactivity at dilutions up to 1∶8000.

### Changes in anti-PLA_2_R reactivity in follow-up samples

To investigate whether anti-PLA_2_R reactivity was different according to the disease activity, we conducted a longitudinal analysis in a subset of 10 of 100 patients with idiopathic MN with available follow-up serum samples and clinical information. Four of 6 patients who had autoantibodies at the time of kidney biopsy went into remission, and the autoantibodies disappeared in 3 of the 4 patients in remission. However, anti-PLA_2_R Abs were persistent in patients who did not go into remission. The anti-PLA_2_R reactivity continued to be negative, regardless of remission status, in all 4 patients who had no autoantibodies at the time of kidney biopsy (Table 5).

**Table 5:** Alteration of anti-PLA_2_R reactivity in follow-up samples (n = 10).

Patient	Initial		Remission	Follow-up	
	uPCR (g/g)	Anti-PLA_2_R reactivity		uPCR (g/g)	Anti-PLA_2_R reactivity
A	12.22	Positive	Yes	1.73	Negative
B	4.25	Positive	Yes	0	Negative
C	7.11	Positive	Yes	3.24	Negative
D	5.48	Positive	Yes	1.57	Positive
E	9.86	Positive	No	11.43	Positive
F	13.17	Positive	No	4.23	Positive
G	5.60	Negative	Yes	0.23	Negative
H	5.38	Negative	Yes	0.51	Negative
I	13.20	Negative	Yes	2.43	Negative
J	5.85	Negative	No	12.97	Negative

Anti-PLA_2_R, anti-phospholipase A_2_ receptor antibody; uPCR, urine protein-creatinine ratio.

## Discussion

We explored the presence of anti-PLA_2_R in Korean patients with idiopathic MN using western blotting, and we demonstrated that 69% of patients had anti-PLA_2_R antibodies in their serum. Additionally, the prevalence of anti-PLA2R reactivity was 80.0% in patients with idiopathic MN and nephrotic range proteinuria. This finding is in agreement with previous reports on patients who had idiopathic MN and were of different races and ethnicities [5], [6], [7], [11]. Although anti-PLA_2_R is a specific marker for idiopathic MN, autoantibodies were detected in 2 of 9 patients with secondary MN in this study. However, predominant glomerular IgG4 deposition, which is a characteristic feature of idiopathic MN [5], [22], [23], [24], was only detected in 2 patients with anti-PLA_2_R, suggesting that idiopathic MN and hepatitis B or malignancy may occur coincidentally in these patients.

Our study also demonstrated that anti-PLA_2_R reflects disease activity in idiopathic MN. The prevalence of anti-PLA_2_R in patients who were diagnosed with idiopathic MN and had not yet received immunosuppressive treatment was higher than in those who had already gone into remission. Additionally, anti-PLA_2_R detected at the time of initial diagnosis disappeared when the patients entered remission, although autoantibodies were still persistently detected in patients who did not go into remission. Moreover, anti-PLA_2_R levels were in direct proportion to the initial clinical values of serum albumin levels and proteinuria. Taken together, these findings support the hypothesis that anti-PLA_2_R is essential to trigger the disease and reflects disease activity. Although similar results have been reported in previous studies [5], [6], controversy remains about the relationship between anti-PLA_2_R reactivity and disease activity because one study showed discrepant results [11]. This debate is derived from several limitations of previous studies. These studies included a small number of patients and different time points between kidney biopsy and serum sampling, and all of these studies were conducted on Caucasians. Our data may reconcile the disputes from previous studies because our study was conducted in a large number of Korean patients with MN who were in a similar phase of the disease course in the diagnostic stage.

Unfortunately, however, our study did not show a significant difference in the clinical outcomes of remission rate or time to remission according to the anti-PLA_2_R levels. There could be several limitations to interpreting the data. First, the overall remission rate was high (67.5%), and the median time to remission was short (2 months). In this circumstance, it is hard to expect a significant difference between groups. Second, the rate of immunosuppressive treatment was also high (67.5%), which made it difficult to observe the clinical course of the disease according to anti-PLA_2_R levels excluding an effect of immunosuppressive treatment. Therefore, we cannot evaluate solely the effect of anti-PLA_2_R on the outcomes. Nevertheless, the present study showed that most of the patients who had immunosuppressive treatment achieved remission in a short time, although they initially had different severities and different levels of autoantibodies. This finding suggests that the effect of immunosuppressive treatment as a therapeutic intervention is so large that anti-PLA_2_R levels cannot influence the clinical outcomes, although the disease activity at the time of diagnosis is significantly related to the levels of anti-PLA_2_R. On the contrary, a recent study of Caucasian patients with idiopathic MN demonstrated that spontaneous remissions occurred less frequently in patients with high antibody titer, whereas total remission rate was not different [25]. The discrepancy regarding the rate of spontaneous remission may result from differences in the demographic and clinical characteristics of the study subjects. However, considering that the overall remission rate was not different across the antibody titers in an entire study population who received immunosuppressive treatment, the findings of the previous study seem to be consistent with our hypothesis that the autoantibody *per se* could not predict the outcome excluding the huge effect of therapeutic intervention.

In previous studies, anti-PLA_2_R antibodies were not detected in all patients with idiopathic MN [5], [6], [7]. A possible explanation was that autoantibodies might be measured in an immunologically inactive stage after spontaneous remission or immunosuppressive treatment because serum samples had not been collected in conjunction with renal biopsy. In contrast, we measured anti-PLA_2_R in serum samples that had been collected at the time of renal biopsy. Nevertheless, anti-PLA_2_R was not detected in approximately 30% of patients with idiopathic MN in this study. This finding suggests that alternative target antigens other than PLA_2_R may contribute to the pathogenesis of idiopathic MN. Additionally, our data demonstrated that anti-PLA_2_R continued to be negative in patients with idiopathic MN who had no autoantibodies at the beginning, regardless of their remission status. This result could support our previous hypothesis. Several investigators reported other possible target antigens involved in the development of idiopathic MN [22], [26], [27], [28]. Additionally, we cannot rule out the possibility that in a quantitative respect, anti-PLA_2_Rs were present at sufficient levels to trigger the disease but were not sufficient to be detected with the current method. In other words, the current method might not be sensitive enough to detect trace amounts of autoantibodies.

The present study has several limitations. First, although we accessed the difference of anti-PLA_2_R levels using western blot semi-quantitatively, we could not measure exact titers of the autoantibody. Second, this study is not feasible for assessing the confounding effect of the treatment regimen on anti-PLA_2_R and clinical outcome because of an uncontrolled study design. The treatment regimen was decided according to the clinician’s preference, which was based on the generally accepted treatment recommendations [19], [29], [30], [31], [32]. However, in some cases, clinicians modified the regimens based on their clinical experiences. The possible heterogeneity in treatment response or adverse effects across the regimens might contribute to the relationship between anti-PLA_2_R titers and clinical outcomes as a confounding factor. Third, the follow-up patients were likely to have more severe clinical manifestations compared with other patients who were lost to follow-up. Thus, the follow-up patients could not represent the initial study population in its entirety, and the outcome from the study would have a limitation when applied to the whole study population. We need additional, well-designed, prospective, randomized studies to exclude these limitations in the near future.

In conclusion, we confirm that anti-PLA_2_R is a specific marker of idiopathic MN in Korean patients, and we hypothesize that the autoantibodies might play a role in triggering the development of the disease. Additionally, the anti-PLA_2_R levels reflect disease activity, with an adverse trend in clinical symptoms according to the antibody levels. Consequently, anti-PLA_2_R could be a useful biomarker for diagnosing MN and monitoring disease activity to predict relapse. However, the present study does not show a significant difference in clinical outcomes, such as remission rate and time to remission, in accordance with anti-PLA_2_R levels, indicating that the autoantibodies may not be a practical marker to predict clinical outcomes or determine therapeutic strategies in current clinical practice.
